# Interstitial fluid flow contributes to prostate cancer invasion and migration to bone; study conducted using a novel horizontal flow bioreactor

**DOI:** 10.1088/1758-5090/acc09a

**Published:** 2023-03-17

**Authors:** Haneesh Jasuja, Sharad V Jaswandkar, Dinesh R Katti, Kalpana S Katti

**Affiliations:** 1 Department of Civil, Construction and Environmental Engineering North Dakota State University, Fargo, ND 58108, United States of America

**Keywords:** prostate cancer, metastasis, bioreactor, shear stress, bone

## Abstract

Prostate cancer bone metastasis is the leading cause of cancer-related mortality in men in the United States, causing severe damage to skeletal tissue. The treatment of advanced-stage prostate cancer is always challenging due to limited drug treatment options, resulting in low survival rates. There is a scarcity of knowledge regarding the mechanisms associated with the effects of biomechanical cues by the interstitial fluid flow on prostate cancer cell growth and migration. We have designed a novel bioreactor system to demonstrate the impact of interstitial fluid flow on the migration of prostate cancer cells to the bone during extravasation. First, we demonstrated that a high flow rate induces apoptosis in PC3 cells via TGF-*β*1 mediated signaling; thus, physiological flow rate conditions are optimum for cell growth. Next, to understand the role of interstitial fluid flow in prostate cancer migration, we evaluated the migration rate of cells under static and dynamic conditions in the presence or absence of bone. We report that CXCR4 levels were not significantly changed under static and dynamic conditions, indicating that CXCR4 activation in PC3 cells is not influenced by flow conditions but by the bone, where CXCR4 levels were upregulated. The bone-upregulated CXCR4 levels led to increased MMP-9 levels resulting in a high migration rate in the presence of bone. In addition, upregulated levels of *α*_v_ *β*_3_ integrins under fluid flow conditions contributed to an overall increase in the migration rate of PC3 cells. Overall, this study demonstrates the potential role of interstitial fluid flow in prostate cancer invasion. Understanding the critical role of interstitial fluid flow in promoting prostate cancer cell progression will enhance current therapies for advanced-stage prostate cancer and provide improved treatment options for patients.

## 1. Introduction

Prostate cancer is the most frequently diagnosed cancer in men that tends to metastasize preferentially to the skeleton, leading to malignant bone lesions. Metastatic prostate cancer cells dysregulate the normal bone remodeling process by distressing bone tissue homeostasis. The 5 year survival rate for localized prostate cancer patients is significantly higher than those with prostate cancer disseminated to a distant organ [1]. To colonize the bone site, tumor cells invade the extracellular matrix (ECM) of the growing tumor at their primary site, intravasate into the blood circulation, and then extravasate from the blood vasculature to the bone ECM. *In vitro* models have been proven beneficial for studying cancer cell progression and developing novel anticancer drugs. In particular, 3D *in vitro* disease models mimic the pathophysiological microenvironment more due to the close interaction between different cell types and the release of factors responsible for the generation of ECM in a precise system [2, 3]. 3D *in vitro* models bridge the gap between 2D monoculture models, which do not possess *in vivo* structural complexity, and expensive *in vivo* models, often failing to recapitulate the late stage of cancers. Previous studies report development of 3D *in vitro* disease models of prostate [4, 5] and breast cancer [6, 7] bone metastasis using polymer-nanoclay-based scaffolds that exhibit high porosity of 86.1% and pore sizes ranging between 10–30 *µ*m and 100–300 *µ*m and possess a compressive modulus of 2.495 MPa [8], required for hard tissue growth. The role of interstitial fluid in prostate cancer progression to the bone using a 3D *in vitro* model with a perfusion bioreactor has also been reported earlier [9]. However, understanding the metastatic cascade of cancer cells is essential to pave the way to discover novel drugs for metastatic cancers. In particular, the extravasation stage is a critical process for cancer cell invasion to the secondary site and subsequent development of metastatic tumors. Extravasation comprises various stages, including slowly rolling, adherence to the sinusoidal capillaries, and transmigration across the capillary’s endothelium [10]. Recently, several groups have developed microfluidic platforms to recapitulate the extravasation microenvironment that majorly focuses on the effects of biomechanical cues on tumor cell motility [11–14]. In addition, Boyden chamber-transwell assays deliver a relatively simple and high throughput system for quantifying percentage cell migration [15], yet do not fully recapitulate extravasation and migration behavior under physiological fluid flow conditions. While these models have provided useful insight into the migration behavior of cancer cells at their distant sites, they did not adequately address the effect of interstitial fluid flow on cancer cells’ migration rate and their molecular mechanisms.

In the present study, we hypothesized that interstitial fluid flow acts as a driving force for cancer cell migration during the extravasation stage. Thus, we developed a novel 3D *in vitro* dynamic model integrated with transwell inserts that recapitulate *in vivo* microenvironment representing the migration of cancer cells under interstitial fluid flow. Here, we evaluate the migration of PC3 prostate cancer cells through transwell insert under both dynamic and static culture conditions. We investigate the molecular mechanisms responsible for the migration of cancer cells under different culture conditions. This study demonstrates that *α*_v_ *β*_3_ integrins play distinct roles in response to mechanical cues and act as mechanosensory that transduce mechanical signals via *α*_v_ *β*_3_-MMP-9 signaling axis to promote flow-induced motility of prostate cancer cells.

## 2. Materials and methods

### 2.1. Scaffold preparation and microCT imaging

Polycaprolactone (PCL)-*in situ* hydroxyapatite (HAP) clay scaffolds were prepared as per the protocol described previously in the literature [8, 16–19]. In brief, we modified sodium montmorillonite (Na-MMT) clay with an amino acid modifier (5-aminovaleric acid) to increase the d-spacing between the clay sheets. Further, we mixed HAP and modified clay to biomineralize HAP into intercalated nano-clay sheet galleries. Then, we dissolved PCL and 10% *in situ* HAP Clay in 1,4-dioxane and subjected the resultant solution to freeze-drying extraction to obtain PCL/*in-situ* HAP Clay scaffolds. We utilized cylindrical scaffolds with dimensions of 12 mm diameter and 3 mm thickness during experiments.

The scaffold samples were scanned using a micro-CT scanner (GE Phoenix vltomel xs x-ray computed tomography system) with an 80 kV x-ray energy source and 350 *μ*A current intensity with a molybdenum target. Scans were performed at multiple detector exposure times of 200 ms, 500 ms, 1000 ms, and 2000 ms, and the final image was reconstructed using a 500 ms detector timing. The sample magnification was carried out with a voxel size of 15.51 *μ*m. The microCT images of the scaffold (supplementary document 2 figure S2) showed high interconnected porosities ranging between 79.4% and 81.65%, necessary for fluid flow through the scaffold, with pores sizes ranging between 10–30 *µ*m and 100–300 *µ*m.

### 2.2. Horizontal flow bioreactor design

**Figure 1.**
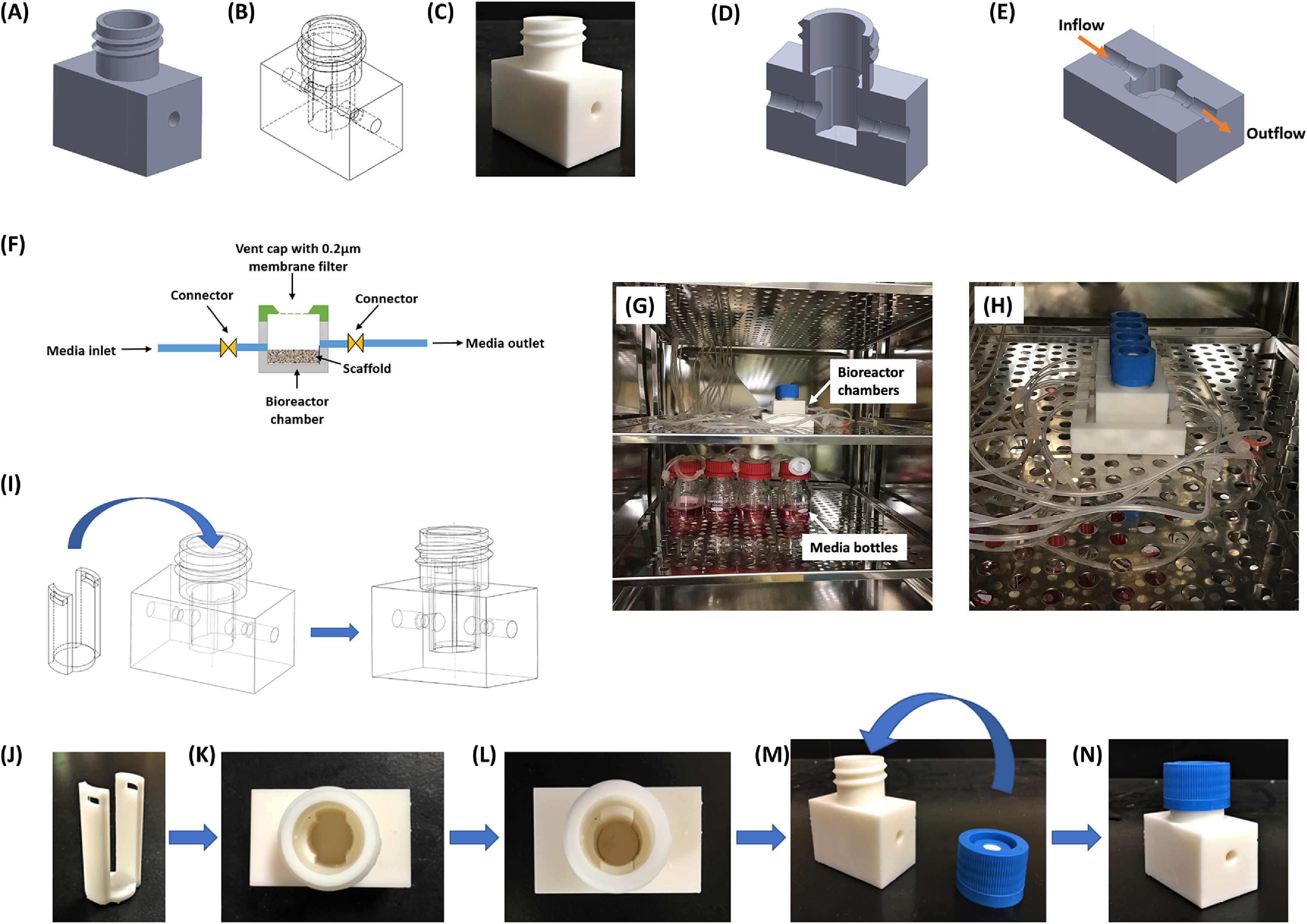
Bioreactor design (A) CAD design of bioreactor chamber (full view) (B) symmetric view of the chamber (C) 3D printed bioreactor chamber (D) section of side view (E) section of top view. Arrow indicates inflow and outflow fluid directions (F) schematic representation of bioreactor and their components with flow directions. (G), (H) Bioreactor assembly setup inside the incubator (37 °C, 5% CO_2_, and high moisture content) (G) bioreactor chambers (top rack) connected to media bottles (bottom rack) via tubing (H) enlarged view of bioreactor chambers positioned inside the incubator. (I) Symmetric view of the chamber and holder showing steps of holder insertion into bioreactor chamber (J)–(N) assembly of bioreactor chamber (J) scaffold holder (K) top view of bioreactor chamber (L) top view of the bioreactor chamber inserted with scaffold holder (M) bioreactor chamber and vent cap (N) vent cap fitted bioreactor chamber.

The horizontal bioreactor chambers are designed with computer-aided design (CAD) software (SolidWorks v.2018, Dassault Systems) and fabricated using biocompatible crosslinked polymethyl-methacrylate polymer (RS-F2-GPWH-04 white resin) by a Formlabs Form 2 3D printer with a resolution of 50 *µ*m. The dimensions of the rectangular culture chamber are 50 mm × 30 mm × 32 mm, and each culture chamber supports one scaffold sample (figures 1(A)–(E)). A scaffold holder is designed to put the scaffolds deep into the culture chambers without flipping (figures 1(I)–(L)). The inlet and outlet of bioreactor chambers are connected to tubing (Peroxide-Cured Silicone, ID 1.42 mm, Ismatec) for continuous inflow and outflow of cell culture medium, respectively (figures 1(F)–(H)). Media containing borosilicate bottles and a flow regulating pump are coupled with bioreactor chambers via these tubing. Experiments were carried out at two different flow rates—0.2 ml min^−1^ and 0.05 ml min^−1^. A vent cap with a 0.2 *µ*m gas permeable membrane is placed on the top of the chamber to cover the opening that facilitates gas exchange within the culture chamber and maintains a sterile environment (figure 1(M) and (N)). The whole assembly is placed inside the incubator (figures 1(G) and (H)). The media was pumped into the bioreactor chamber’s inlet via a flow-regulating pump. Media enters the bioreactor chamber and flows through the scaffold, penetrates porous microstructures, and exits the chamber through the outlet.

### 2.3. Computational fluid dynamics (CFDs) analysis

The bioreactor model and the transwell insert were designed using CAD software (SolidWorks v.2018, Dassault Systems). The theoretical analysis of the hydrodynamic performance of the bioreactor was conducted by CFD using a SolidWorks flow simulation package. The SolidWorks Flow Simulation is a CAD-integrated CFDs simulation software fully integrated with the part design environment [20]. It is based on a Cartesian meshing approach integrated directly into the native CAD system [20]. The software uses a discrete numerical technique based on the finite volume method to integrate with CFD solvers. The fluid medium in the bioreactor was assumed to be an incompressible fluid with a dynamic viscosity of 6.9 × 10^−4^ Pa·s and a density of 993.3 kg m^−3^ (dynamic viscosity and density of water at 37 °C). The scaffold geometry was considered solid with dimensions of 12 mm diameter and 3 mm thickness, and the total porosity assigned was 81%, determined from microCT images of the scaffold (supplementary document 2 figure S2). The inlet flow rates of 0.05 ml min^−1^ and 0.2 ml min^−1^ were applied to the bioreactor model based on the literature [10, 13, 14]. The permeability of a porous media in the form of medium resistance to fluid flow (*k*) was defined as $k = \frac{{32\mu }}{{\varepsilon \delta {D^2}}}$ where *µ* and *δ* are the fluid dynamic viscosity and density, respectively, *D* is the pore size, and *ϵ* is the porous medium’s porosity. The mesh sensitivity analysis was performed to determine the optimum number of fluid cells required to simulate fluid dynamics accurately. The parameters used for the system are described in table 1.

**Table 1.** CFD parameters and approximations applied for the bioreactor system.

Temperature (°C)	37
Pressure (Pa)	101 325
Gravitational constant (m s^−2^)	−9.81
Fluid	Water
Flow type	Laminar and turbulent
Wall thermal condition	Adiabatic
Boundary conditions	Inlet and outlet at a uniform flow rate
Mesh	Global mesh refinement: 4 local mesh refinement: 5
**Porous media**	
Porosity	0.81
Permeability type	Isotropic
Resistance calculation formula	$k = \frac{{32\mu }}{{\varepsilon \delta {D^2}}}$
Pore size (*µ*m)	25 *µ*m

### 2.4. Cell lines and cell culture

Human mesenchymal stem cells (hMSCs) (PT-2501) (Lonza) were maintained in a complete growth medium (MSCGM Bulletkit medium (Lonza, PT-3001)). Human prostate cancer cells-PC3 (ATCC® CRL-1435™) were purchased from American Type Culture Collection (ATCC) and maintained in complete growth medium (Kaighn’s Modification of Ham’s F-12 Medium-ATCC, 30-2004, 10% Fetal Bovine Serum (ATCC, 30-2020), and 1% Pen-Strep antibiotic (Gibco)). The cell cultures were maintained at 37 °C and 5% CO_2_ in a humidified incubator.

### 2.5. Cell seeding

We first sterilized the scaffolds under ultraviolet light for about 1 h and then immersed the scaffolds into 70% ethanol solution for 12 h. Further, we washed the scaffolds twice with 1X phosphate buffer saline (PBS). We seeded 1 × 10^5^ PC3 cells on each scaffold for flow-optimization-related studies, including DNA quantification assay, live-dead assay, cell apoptosis, western blot, and gene-expression experiments unless otherwise specified. For migration studies, we seeded 5 × 10^5^ hMSCs on each scaffold under static culture and allowed them to osteogenically differentiate into osteoblasts resulting in mineralized bone formation at 23 d, as reported previously [7, 21]. After 23 d, we transferred hMSCs containing scaffolds into the bioreactor and seeded 4 × 10^4^ PC3 cancer cells into each transwell insert. The detail of the migration experiment is provided in section 2.10. We refreshed the media for the bioreactor samples every 2 d and static samples every other day.

### 2.6. DNA quantification

DNA quantification assay was performed to assess the proliferation rate of PC3 cells by measuring their DNA content using a kit (AccuBlue® Broad Range dsDNA Quantitation Kits). We followed the procedure described elsewhere for sample preparation [9]. Standard solutions were prepared as per the manufacturer’s protocol. Briefly, 10 *µ*l of each standard and diluted sample was mixed with 200 *µ*l of working solution and incubated for 30 min at room temperature in the dark. The fluorescence was measured at Ex350 nm/Em460 nm using a fluorescence microplate reader (BioTek).

### 2.7. Live dead assay

A live-dead assay was performed to evaluate the viability of PC3 cells under static and flow conditions. The scaffolds seeded with PC3 cells were introduced with both live (Calcein AM) and dead (Ethidium Homodimer III (EthD-III)) stains at different time points (Day-4 and Day-8) using a standard protocol (Biotium, 30002-T). Briefly, the scaffolds were rinsed twice with PBS and were incubated in 2 *µ*M calcein AM and 4 *µ*M EthD-III in PBS for 30 min at room temperature. Next, the scaffolds were imaged under Zeiss Axio Observer Z1 LSM 700 confocal microscope using Ex/Em wavelengths described in the manufacturer’s protocol. We imaged different regions of the scaffold while performing the live-dead assay. The presented live-dead images in the manuscript are obtained from the areas of the scaffolds where we expected maximum variability due to high shear stress as predicted from CFD analysis.

### 2.8. Cell apoptosis by flow cytometry

The apoptosis analysis was carried out using the propidium iodide (PI)-Annexin V double staining method per the standard procedure (Biolegend). Briefly, samples were retrieved from different conditions on Day 4 and Day 8 and washed thoroughly with cold PBS. Cancer cells were harvested from each scaffold by treating them with 500 *μ*l of TrypLE™ Express enzyme. Next, cancer cells were resuspended in Annexin binding buffer to 1 × 10^6^ cells ml^−1^ concentration. Further, 100 *μ*l of cell suspension of each condition was treated with 5 *μ*l of fluorescein isothiocyanate conjugated Annexin V and 10 *μ*l of PI stains and incubated in the dark for 15 min at room temperature. The cell suspension of each sample was further diluted with 400 *μ*l of Annexin binding buffer and analyzed using a BD Accuri C6 Flow cytometer.

### 2.9. Gene expression by RT-qPCR

Scaffold samples containing PC3 cells were retrieved from different culture conditions on Day 8 to assess their apoptosis-related gene expressions. RNA was isolated using TRIzol reagent and purified using a Direct-zol RNA MiniPrep kit (Zymo Research). For migration-related gene expressions, PC3 cells were treated directly on a transwell membrane with TRIzol reagent after 24 h of the migration experiment for their RNA isolation. Next, RNA was reverse transcribed to cDNA using random primers and M-MLV reverse transcriptase (Promega), and the mRNA expressions were quantified using SYBR green master mix. The qPCR reaction conditions used during each run include holding stage—95 °C, 5 min, followed by cycling stage—40 cycles of 95 °C, 30 s, and 60 °C, 1 min. The expressions of various genes related to apoptosis and migration TGF*β*-1, Caspase-9, Bcl-2, p53, *α*_v_, *β*_3_, MMP-9, and CXCR4 were analyzed and normalized to the mean of *β* actin. The details of the primers are given in supplementary document 1 table S1. The relative fold change was calculated using the 2^(−$\Delta \Delta $Ct) comparative method.

### 2.10. Transwell migration assay

For the transwell migration assay, we have customized the bioreactor assembly to fit the transwell insert better and accommodate bone mimetic scaffolds underneath the transwell insert. A total of 4 × 10^4^ PC3 cancer cells in 100 *μ*l PC3 media containing 2% FBS were seeded into each transwell insert of 8.0 *μ*m pore size (Corning, Inc., Corning, NY, USA) and allowed to adhere to the surface for 3 h. Next, the media was replaced with 100 *μ*l serum (FBS) free media, and the inserts were moved into the bioreactor. The cells were allowed to migrate towards the lower chamber containing complete PC3 media (F-12 K with 10% FBS) and towards tissue-engineered bone (osteogenically differentiated hMSCs containing scaffold described in section 2.5) with complete PC3 media (F-12 K with 10% FBS). After 24 h of incubation, PC3 cells that remained on the upper surface of the transwell membrane were gently removed using cotton swabs shown in the schematic (supplementary document 2 figure S3). The percentage of cells that migrated through the transwell membrane and adhered to the lower surface was measured using Alamar Blue reagent assay (Invitrogen) following the manufacturer’s protocol. The fluorescence intensity was measured using excitation 570 nm and emission 600 nm. PC3 cells seeded in the wells without inserts were considered positive control, and fluorescence emitted only by Alamar blue reagent was regarded as a negative control or background signal. The percentage migration was calculated by using the formula

\begin{align*}{\text{Percentage migration }} = {\text{ }}\frac{{{\text{Fluorescence of migrated cells}} - {\text{background signal}}}}{{{\text{Fluorescence of total cells without insert}} - {\text{background signal}}}}\times{\text{ }}100\end{align*}

### 2.11. Western blot analysis

PC3 cells were harvested from scaffolds on Day 8, and protein was extracted using RIPA lysis buffer. Next, total protein was estimated using the Bradford assay (Thermofisher). The proteins were separated using 10% (v/v) SDS-PAGE gels and transferred to 0.2 *µ*m PVDF membrane. The membrane was blocked for 1 h at RT with a blocking buffer (5% bovine serum albumin, 0.05% Tween-20 Alfa Aesar). Next, the membrane was incubated with a primary antibody overnight at 4 °C. The primary antibodies used for the analysis were p-Smad2 (Cell signaling #3108, 1:1000 dilution), Smad2 (Cell signaling #3102, 1:1000 dilution), p-Akt1 (Cell signaling #9271, 1:1000 dilution), and Akt (Cell signaling #9272, 1:1000 dilution). Further, the membrane was incubated for 1 h at room temperature with a horseradish peroxidase-conjugated secondary antibody at 1:5000 dilution. The blots were scanned under a chemiluminescence imaging system (Applied Biosystems).

### 2.12. Statistical analysis

GraphPad Prism v7.04 software was used to perform statistical analysis. The data were presented as mean ± standard deviation (SD). Data were analyzed using one-way, or two-way ANOVA followed by Tukey’s post hoc analysis. The difference between the two groups was considered as statistically significant for *p* < 0.05.

## 3. Results

### 3.1. Optimization of interstitial flow velocities and shear stress for optimum cell growth

**Figure 2.**
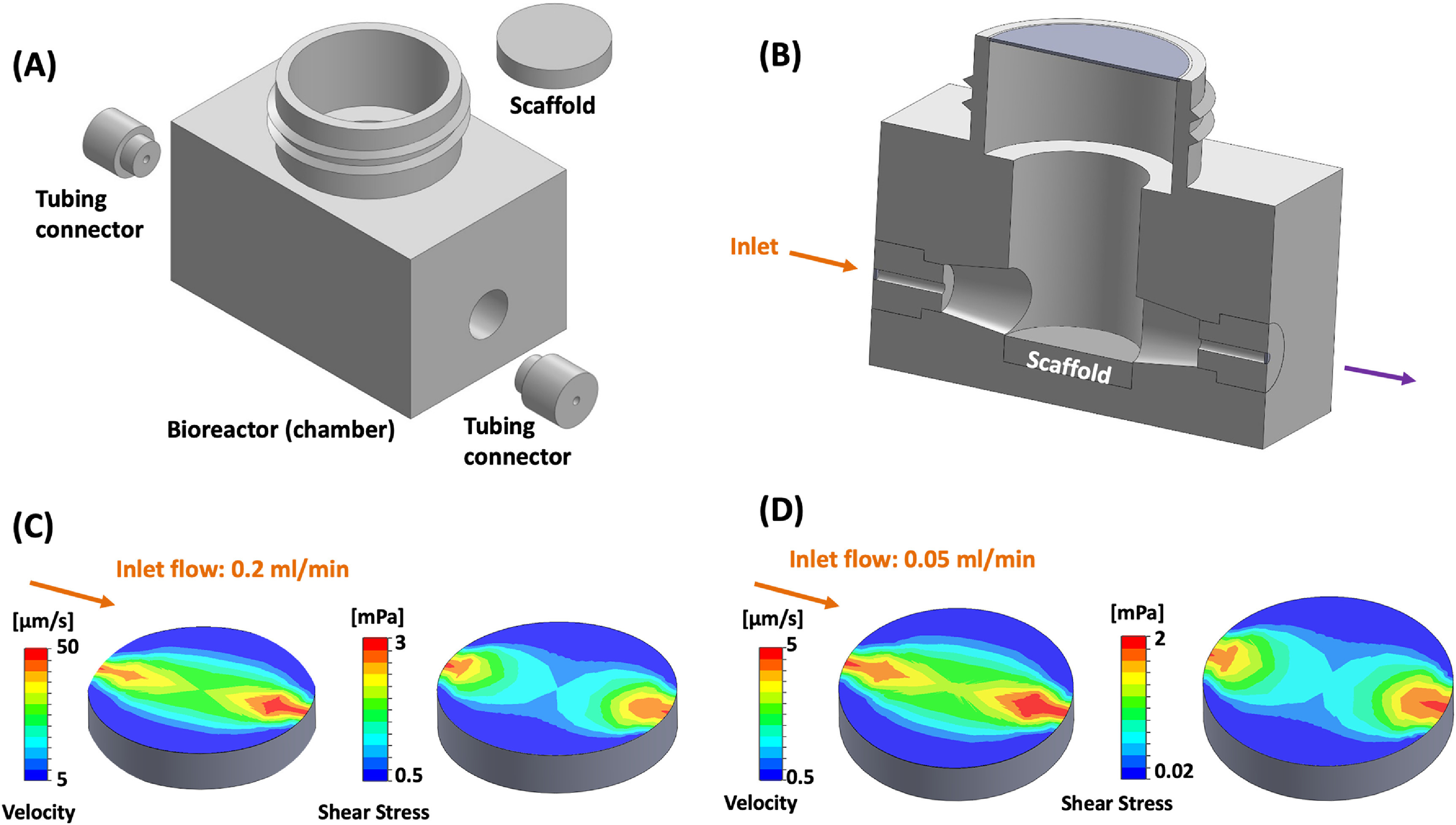
(A) Bioreactor assembly components (B) sectional view of bioreactor assembly (C) velocity and shear stress distribution on scaffold surface at inlet flow of 0.2 ml min^−1^ (D) velocity and shear stress distribution on scaffold surface at inlet flow of 0.05 ml min^−1^. Arrows represent the direction of fluid flow.

The reported interstitial fluid velocities range *in vivo* is 0.1–4 *μ*m s^−1^ [12, 13]. Here, we analyzed two different inlet flow rates—0.05 ml min^−1^ (low flow rate) and 0.2 ml min^−1^ (high flow rate) —to attain physiological interstitial fluid velocity range and understand the correlation between fluid shear stress and cellular response. As we seeded cells on the scaffolds’ top surface, we targeted attaining physiological velocity on the top surface. However, we also analyzed flow velocity distribution at the scaffold’s different sections (vertical and horizontal) (supplementary document 2 figure S1) at 0.05 ml min^−1^ flow rate. The simulated fluid flow through the scaffold analyzed by CFD-displayed heterogeneous fluid velocity. The velocity range attained at different flow rates on the scaffold surface were 5.0 *μ*m s^−1^ (vel_min_) and 50 *μ*m s^−1^ (vel_max_), and 0.5 *μ*m s^−1^ (vel_min_) and 5.0 *μ*m s^−1^ (vel_max_) corresponding to 0.2 ml min^−1^ and 0.05 ml min^−1^, respectively. However, in different horizontal and vertical sections of the scaffold, the flow velocity ranged between 0.0001 *μ*m s^−1^–0.1 *μ*m s^−1^ and 0.0001 *μ*m s^−1^–2 *μ*m s^−1^, respectively. The fluid shear stress estimated for 0.2 ml min^−1^ on the top surface ranged between 0.5 mPa and 3 mPa, while 0.05 ml min^−1^ ranged between 0.02 and 2 mPa. The color gradient indicates faster flow in the scaffold’s center than the stagnant fluid layer near the wall (figures 2(A)–(D)). From CFD results, we concluded that the physiological velocity was achieved on the scaffold surface at a 0.05 ml min^−1^ flow rate and the estimated proportion of the total flow that passes through the scaffold is 31.43%. However, we performed a cell viability assay using both flow rates to understand the effect of a high flow rate on cellular response and to optimize the flow rate experimentally.

### 3.2. High flow rate inhibits cell growth and induces apoptosis

**Figure 3.**
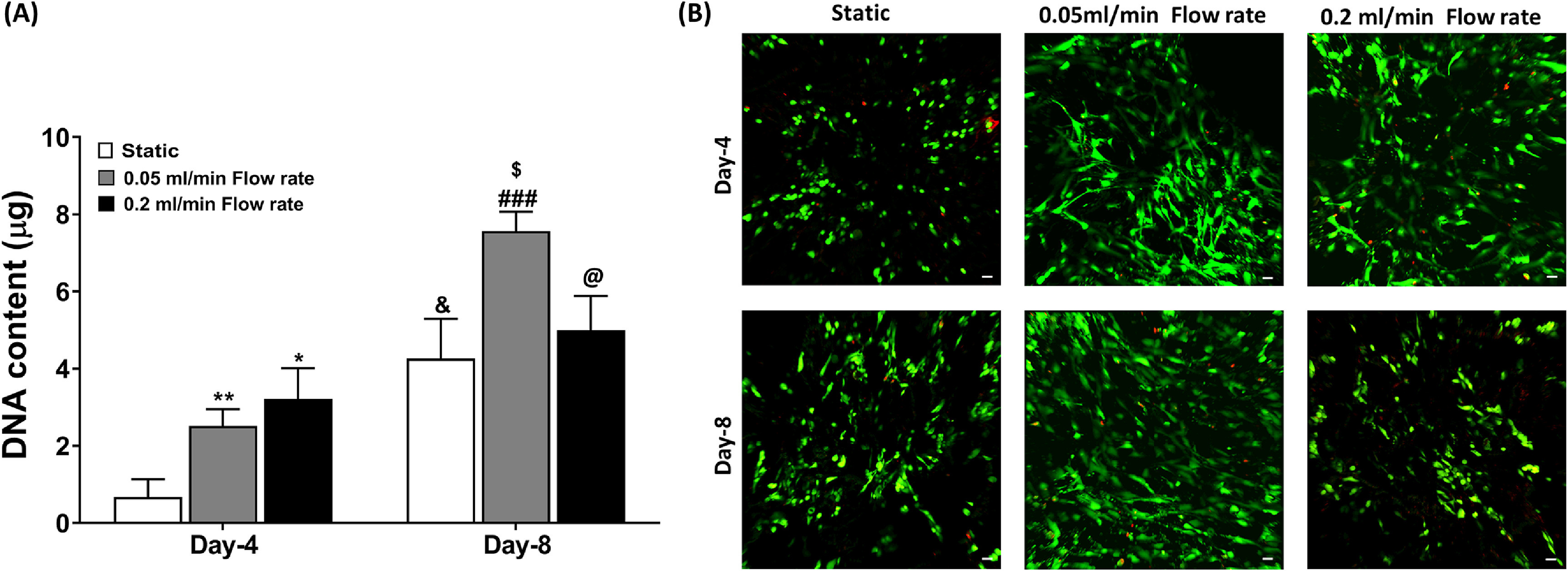
Cell viability was assessed on Day 4 and Day 8 using Static culture (control), 0.05 ml min^−1^ flow rate, and 0.2 ml min^−1^ flow rate (A) DNA content (B) live-dead assay. Green fluorescence represents live cells, and red fluorescence represents dead cells. Scale bar 100 *μ*m *p < 0.05 and **p < 0.01 indicates a significant difference between the static sample and different flow rates samples on Day 4. ^&^ *p* < 0.05 indicates a significant difference between the static sample on Day 4 and Day 8. ^###^ *p* < 0.001 indicates a significant difference between 0.05 ml min^−1^ flow rate sample at Day-4 and Day-8. ^$^ *p* < 0.05 indicates a significant difference between the static sample on Day 8 and the 0.05 ml min^−1^ flow rate sample on Day 8. ^@^ *p* < 0.05 indicates a significant difference between the 0.05 ml min^−1^ flow rate sample on Day 8 and 0.2 ml min^−1^ flow rate sample on Day 8.

To assess the viability of PC3 cells under static and different flow conditions, we measured their DNA content. We observed that the DNA content of PC3 cells on Day 4 was significantly higher under both 0.2 ml min^−1^ (*p < 0.05) and 0.05 ml min^−1^ (**p < 0.01) flow conditions compared to static culture. Similarly, on Day 8, we observed significantly higher DNA content of PC3 under 0.05 ml min^−1^ (^$^ *p* < 0.05) compared to their static culture. However, the DNA content of PC3 cells under 0.2 ml min^−1^ flow rate was significantly decreased (^@^ *p* < 0.05) compared to 0.05 ml min^−1^ flow rate conditions (figure 3(A)). Live-dead staining assay revealed similar outcomes, representing a reduced population of live cells under high flow rate conditions on Day 8 compared to low flow rate conditions (figure 3(B)). In addition, we observed a significant difference in PC3 cell growth within different regions of the scaffold. We noticed poor cell growth in the center of the scaffold, where cells encountered high shear compared to the sides of the scaffold, where cells were growing under low shear, predicted by CFD analysis.

### 3.3. *TGF-β*1 induces apoptosis under a high flow rate

**Figure 4.**
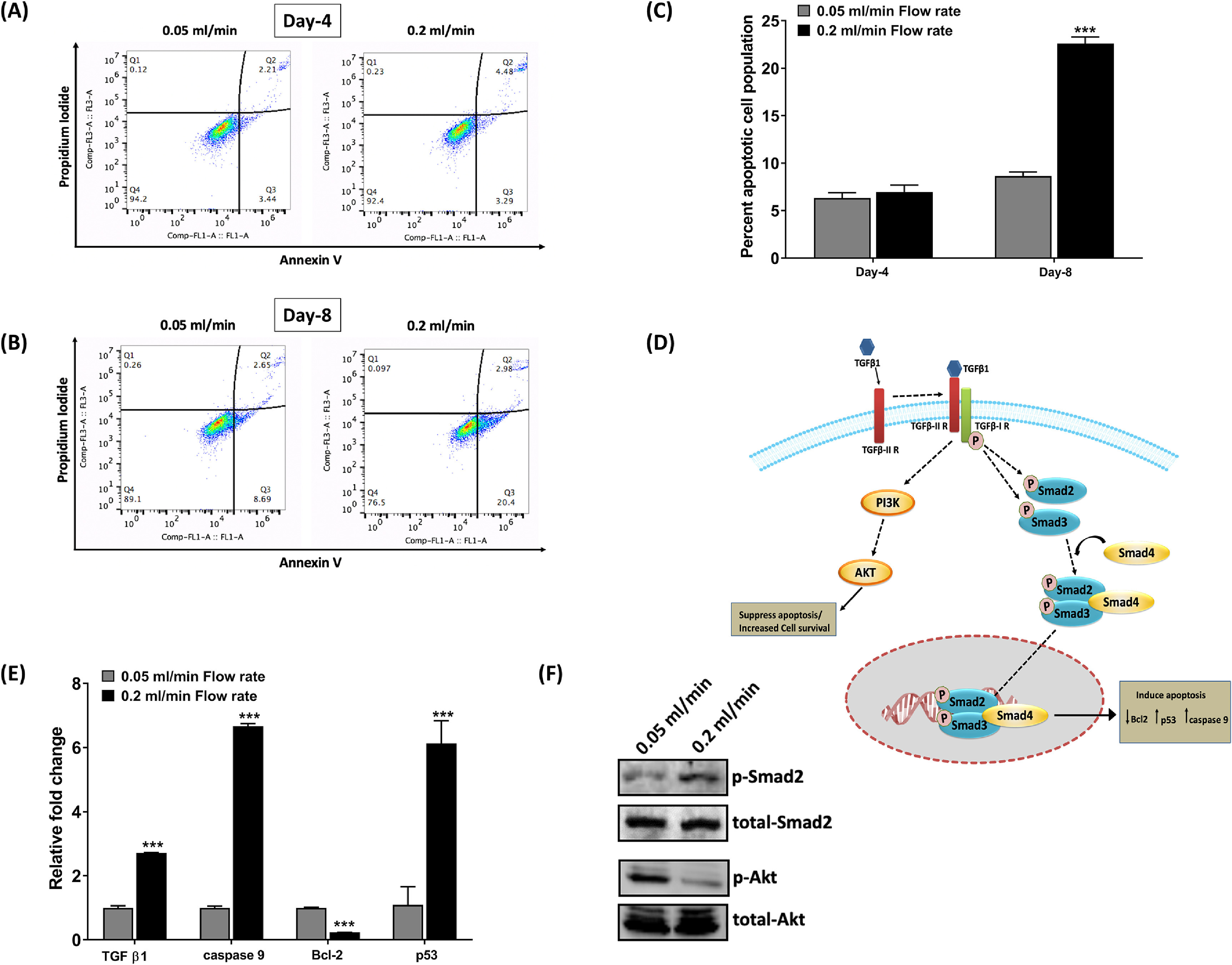
Cell apoptosis was assessed at 0.05 ml min^−1^ and 0.2 ml min^−1^ flow rates (A) and (B) representative dot plot presenting percent live, early, and late apoptotic cells following double staining with Annexin V and propidium iodide on Day 4 and Day 8. (C) The percentage of cell apoptosis was calculated on Day 4 and Day 8 by adding percentage early apoptotic and late apoptotic cells under different conditions. (D) Possible mechanism of TGF-*β*1 mediated tumor suppression and tumor induction. (E) Quantitative RT-PCR data for apoptosis-related genes on Day 8. (F) Protein expression of p-Smad2 and p-Akt was assessed by western blotting on Day 8. ***p < 0.001 indicates a significant difference between the samples at 0.05 ml min^−1^ and 0.2 ml min^−1^ flow rates.

To investigate the possible reason for decreased cell growth of PC3 cells under a high flow rate, we performed a cell apoptosis assay by flow cytometry. We did not observe significant changes in the apoptosis rate on Day 4 between 0.2 ml min^−1^ and 0.05 ml min^−1^ flow rates; however, on Day 8, we observed a significantly higher percentage of apoptotic cells (22.60 ± 0.55%) cultured under a high flow rate compared to low flow rate conditions (11.01 ± 0.50%), indicating 0.2 ml min^−1^ flow rate is not suitable for PC3 cells growth for a prolonged period (figures 4(A)–(C)).

Next, we examined the apoptosis-related genes to investigate the molecular mechanism responsible for the apoptotic induction of PC3 cells at Day 8 under a high flow rate. We observed that mRNA levels of the tumor suppressor gene, p53 (***p < 0.001), and apoptotic gene, caspase-9 (***p < 0.001), were significantly upregulated in PC3 cells under a high flow rate. In contrast, expressions of the antiapoptotic gene, Bcl-2 (***p < 0.001), were downregulated, suggesting apoptotic induction in PC3 cells under a high flow rate. We also examined mRNA levels of TGF-*β*1, which is highly accountable for apoptotic induction in tumor cells [22]. We observed a significant upregulation in TGF-*β*1 mRNA levels (***p < 0.001) under high flow rate compared to low flow rate conditions. It is also reported that TGF-*β*1 acts as both a tumor suppressor and tumor inducer, promoting cell apoptosis via the Smad-dependent pathway while suppressing apoptosis or enhancing cell survival via the Smad independent-PI3K/Akt pathway [23]. Thus, we decided to investigate the feasibility of these two signaling pathways to understand the possibility of an increase in TFG-*β*1 mRNA levels at high flow rate conditions. We observed significantly high protein expression of p-Smad2 under high flow rate conditions compared to low flow rate conditions, indicating the tumor suppressor effect of TGF-*β*1 under high flow conditions. In addition, we observed a thick band of p-Akt under low flow conditions and a very thin p-Akt band under high flow rate conditions, suggesting that the survival of PC3 cells was decreased under high flow conditions (figures 4(D)–(F)).

### 3.4. Physiological interstitial fluid velocity induces a high migration rate of prostate cancer cells

**Figure 5.**
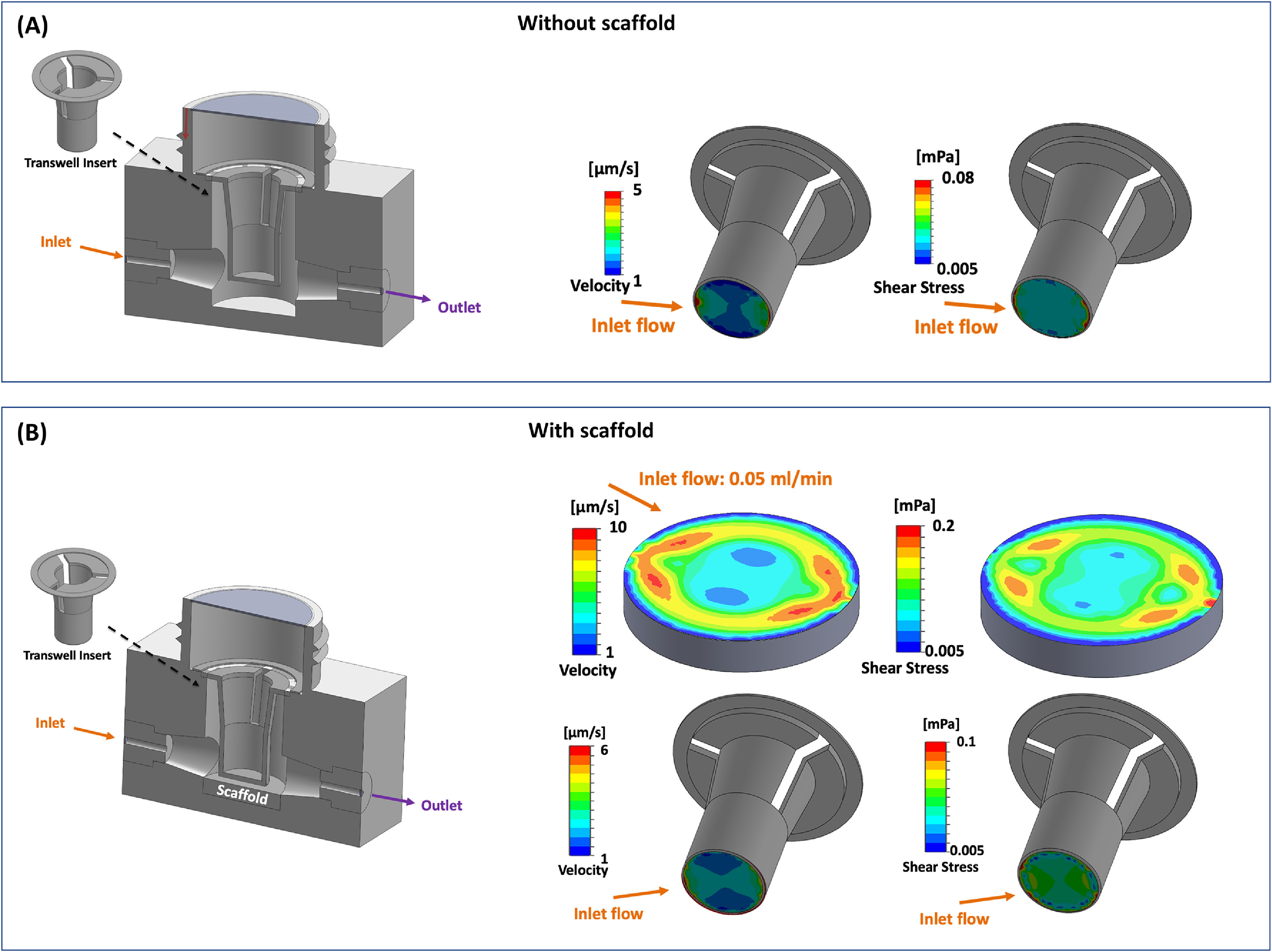
(A) Bioreactor assembly with transwell insert without scaffold (upper left), velocity and shear stress distribution on transwell membrane surface at inlet flow of 0.05 ml min^−1^ (upper right). (B) Bioreactor assembly with transwell insert and scaffold (lower left), velocity and shear stress distribution on the scaffold and transwell membrane surface at inlet flow of 0.05 ml min^−1^ (lower right). Arrows represent the direction of fluid flow.

Next, to evaluate the migration of PC3 cells through transwell insert under flow conditions, we employed a 0.05 ml min^−1^ flow rate based on the CFD (figure 2) and viability (figure 3) results. From CFD analysis using a transwell insert without scaffold (figure 5(A)), we demonstrated that shear stress attained at the membrane of the transwell insert ranged between 0.005 mPa and 0.08 mPa, which was nearly equivalent to the shear stress range (0.005 mPa–0.1 mPa) at the membrane of transwell insert in the presence of scaffold (figure 5(B)), indicating that similar fluid derived shear stress acting on PC3 cells in the presence or absence of tissue-engineered bone. We also analyzed the flow velocity range at the membrane of transwell insert (figures 5(A) and (B)) and in-between the transwell insert bottom and scaffold surface which specifies that the fluid velocity lies well within physiological range of interstitial fluid flow.

**Figure 6.**
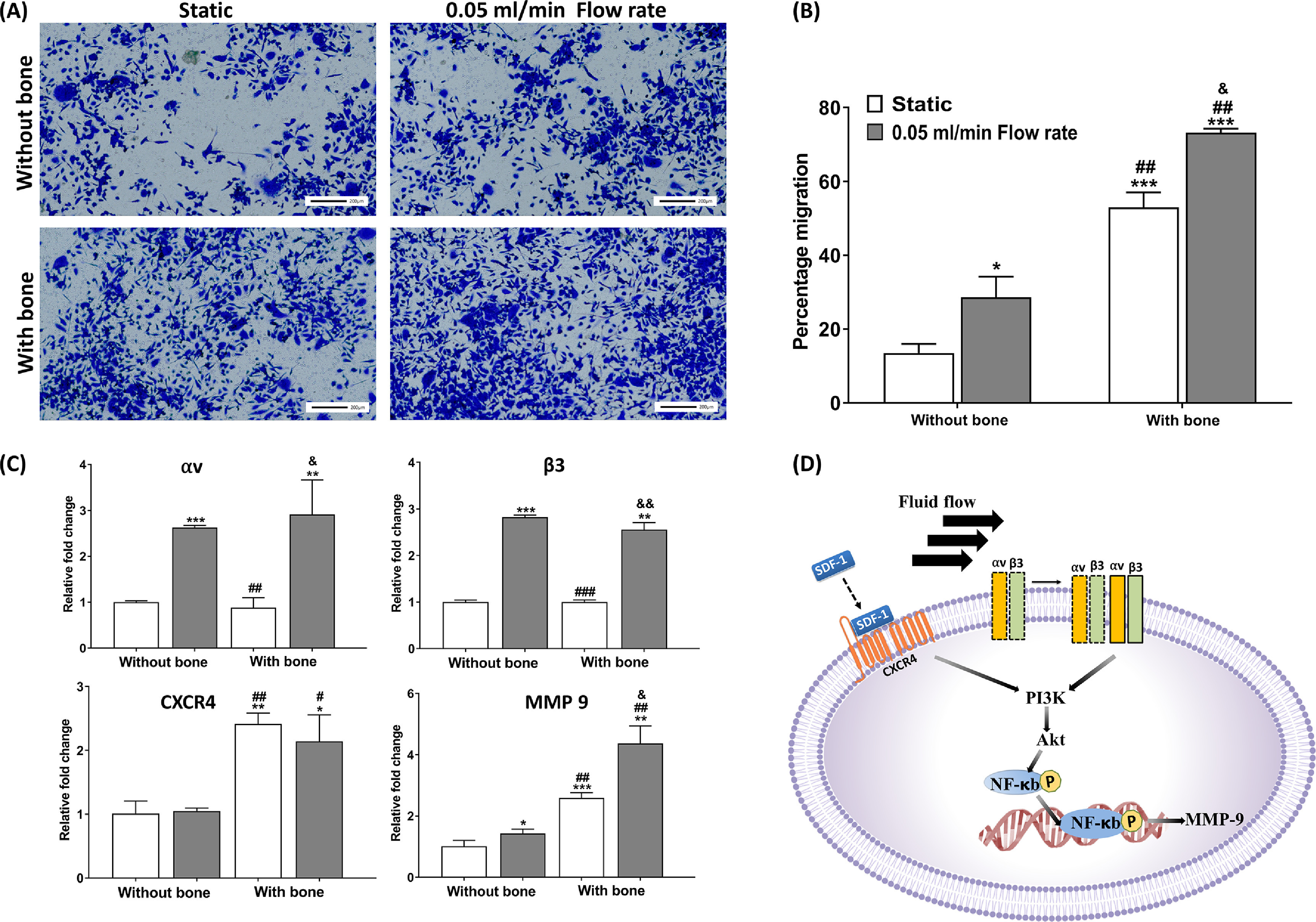
Transmembrane invasion assay showing migration of PC3 cancer cells through transwell inserts with and without bone under static and dynamic culture. (A) Migrated cells stained with crystal violet dye. (B) Percentage cell migration determined using Alamar Blue assay. (C) Gene expression of migration-related genes determined by RT-qPCR. (D) Proposed mechanism of CXCR4 and *α*_v_ *β*_3_ integrins mediated increase in MMP-9 levels under dynamic conditions in the presence of bone. *p < 0.05, **p < 0.01, and ***p < 0.001 indicates a significant difference between the static sample without bone and other conditions. ^#^ *p* < 0.05 and ^##^ *p* < 0.01 indicates a significant difference between the dynamic sample without bone and other conditions. ^&^ *p* < 0.05 indicates a significant difference between the static sample with bone and the dynamic sample with bone.

Experimentally, first, we evaluated the migration rate of PC3 cells under static and dynamic conditions without placing a bone-containing scaffold underneath the transwell insert to understand the effect of continuous fluid flow on migration rate. After 24 h of incubation, we observed that the percentage migration of PC3 cells under fluid flow was increased by ∼2-fold (28.64 ± 5.61%) compared to cells under static condition (13.51 ± 2.47%), indicating the effect of fluid flow on the migration rate of PC3 cells. Next, we evaluated the effect of flow conditions on cell migration rate in the presence of bone. We observed that the overall percentage migration through the transwell insert was increased in the presence of tissue-engineered bone under both culturing conditions; however, under dynamic conditions, the percentage of cell migration (73.24 ± 1.05%) was significantly higher (^&^ *p* < 0.05) than static culture (52.95 ± 4.08%) (figures 6(A) and (B)). Thus, we examined migration-related gene expressions under different culture conditions to investigate the molecular mechanism responsible for the observed change in percentage cell migration under flow conditions and in the presence of bone.

### 3.5. *α*_v_ *β*_3_ integrins activation *via* fluid flow promotes percentage cell migration

Fluid shear stress activates *α*_v_ *β*_3_ integrins that convert mechanical stimulation into chemical signals inside the cells and activate downstream signals [24–27]. We observed that the mRNA levels of *α*_v_ (***p < 0.001) and *β*_3_ (***p < 0.001) integrins in PC3 cells were significantly upregulated under dynamic conditions compared to static conditions in the absence of bone. Next, we investigated MMP-9 gene expressions and observed that mRNA levels of MMP-9 were also significantly upregulated under dynamic conditions (*p < 0.05) compared to static conditions in the absence of bone, indicating the effect of fluid shear stress on cancer cell migration. We also demonstrated mRNA levels of *α*_v_, *β*_3_, and MMP-9 in PC3 cells in the presence of tissue-engineered bone under both dynamic and static culture conditions. The results showed that mRNA levels of *α*_v_ and *β*_3_ in PC3 cells in the presence of bone were not significantly different from those without bone samples. However, expressions of MMP-9 in PC3 cells were significantly higher in the presence of bone than in the absence of bone, indicating the involvement of other factors in the overall increase in cell migration (figure 6(C)). Thus, to understand the reason behind the upregulation of MMP-9 levels in the presence of bone, we planned to investigate other genes related to migration.

### 3.6. CXCR4/CXCL12 interaction leads to increased percent cell migration in the presence of bone

CXCR4 is a crucial regulator of prostate cancer invasiveness and metastasis development [28, 29]. High CXCR4 expression in prostate cancer cells is associated with their propensity to metastasize to the bone, a tissue that expresses a high level of the chemokine CXCL12 [29, 30]. As the results showed upregulation in MMP-9 levels in the presence of bone, we hypothesized that CXCR4 activation led to increased MMP-9 levels. Thus, we investigated CXCR4 mRNA levels of PC3 cells in the presence and absence of bone under both static and dynamic conditions. We observed a significant increase in CXCR4 levels in the presence of bone under both static and dynamic conditions compared to those without bone, indicating that CXCR4 activation in PC3 cells is influenced by the bone (figure 6(C)). We also noticed that CXCR4 mRNA levels were not affected significantly in the presence of flow under both bone and without bone conditions, indicating that CXCR4 activation in PC3 cells is not influenced by the fluid flow (figure 6(C)).

## 4. Discussion

This study investigates the role of interstitial fluid flow in prostate cancer cell migration and the underlying molecular mechanism. Previous studies have shown that interstitial fluid flow has a critical impact on the progression of prostate cancer cells at the bone site [9]. Previously, we explored the role of interstitial fluid flow on hMSCs and prostate cancer cell growth by designing and developing a perfusion bioreactor using a 3D printing technique that allows the media to flow through the scaffold pores. We observed an increased proliferation and differentiation rate of hMSCs under the flow conditions compared to static conditions. In addition we observed that continuous interstitial fluid flow altered the morphology of hMSCs and prostate tumors by altering their gene levels, leading to flattened morphology of hMSCs and compact morphology of tumors under dynamic conditions [9].

However, to understand the role of interstitial fluid flow on the migration of prostate cancer cells at their extravasation stage, customizing the perfusion bioreactor was difficult. Thus, we have designed a new bioreactor model that accommodates transwell inserts for migration studies. Extravasation is a critical step of cancer metastasis, where cancer cells transmigrate from capillaries to a distant organ [10]. Some recent studies have shown the crucial role of interstitial fluid flow in cancer cell motility at a distant organ using microfluidic chip models [13, 14]. However, in the present study, we studied the role of interstitial fluid flow in promoting the migration of prostate cancer cells through transwell inserts. To recapitulate this scenario, we designed a bioreactor system that accommodates both—transwell inserts and tissue-engineered bone, allowing cancer cells to migrate toward bone under interstitial fluid flow. However, before performing this experiment, we optimized the flow rate for cell growth by evaluating their cell viability at different flow rates. We observed a decrease in the DNA content of PC3 cells under a high flow rate (0.2 ml min^−1^) compared to low flow rate (0.05 ml min^−1^) conditions over time, suggesting that a high flow rate induces cell apoptosis (figure 3(A)). TGF-*β*1 has been well explored for its role in inducing cell apoptosis [22, 23, 31]. It is generally suggested that TGF-*β*1 induces cell apoptosis via canonical Smad signaling (figure 4(D)), where TGF-*β*RI and TGF-*β*RII form a complex in the presence of TGF-*β*1 and activates Smad2 and Smad3, which in turn activates Smad4 and thereby mediating programmed cell death via activating pro-apoptotic genes such as caspase 9 [32]. TGF-*β*1 also promotes apoptosis by inhibiting expressions of the antiapoptotic gene, Bcl-2 [33]. We also explored the gene expression of TGF-*β*1 and other apoptosis-related genes. We observed that levels of Bcl2 were downregulated while levels of caspase 9 and p53 were upregulated under high flow rate conditions compared to static culture (figure 4(E)). PI3K/Akt signaling pathway is an essential pro-survival pathway that protects cells against apoptosis-inducing effects of TGF-*β*1 [34, 35]. Thus, a shift in the balance between two different signaling pathways decides the fate of cells. In the present study, we observed upregulation in TGF-*β*1 in high flow rate conditions compared to low flow rate. We also demonstrated hyperactivation of Smad2 under a high flow rate compared to low flow conditions while a decrease in p-Akt levels, suggesting that a shift in balance occurs at high flow rate conditions, leading to cell apoptosis (figure 4(F)).

Based on these results, we decided to carry out migration studies at low flow rate conditions. The *α*_v_ *β*_3_ integrins play a critical role in prostate cancer metastasis to bone. Several studies have confirmed *α*_v_ *β*_3_ integrins mediated adhesion and migration of cancer cells that activate downstream PI3K/Akt signaling, leading to increased cell migration [27, 36, 37]. In the present study, we evaluated the effect of fluid shear stress on *α*_v_ *β*_3_ integrins and MMP-9 levels. We observed that mRNA levels of MMP-9 and *α*_v_ *β*_3_ integrins were upregulated under flow conditions both in the presence and absence of bone, suggesting the role of fluid shear stress on PC3 cell migration via αvβ3 integrins activation, resulted in increased MMP-9 levels (figure 6(C)). However, we also observed that the overall migration rates and MMP-9 levels of PC3 cells were higher in the presence of tissue-engineered bone. Thus, we investigated CXCR4 gene expressions (figures 6(B) and (C)).

CXCR4 is highly expressed in several malignant tumors, including prostate cancer, that confers a more aggressive behavior of cancer cells [38]. CXCL12 (SDF-1)/CXCR4 interactions play an essential role in prostate cancer migration and invasion to the bone by activating Akt1 and MMP-9 expressions [28, 29]. We also observed that CXCR4 levels were significantly higher in the presence of bone compared to those without bone conditions. Previously, it was also reported that interstitial fluid flow increased glioma [39] and hepatocellular carcinoma cell [40] invasion via CXCR4-dependent signaling by increasing CXCR4 levels. However, we did not observe any significant change in CXCR4 levels under dynamic conditions compared to static conditions in the presence or absence of bone (figure 6(C)). Thus, the observed results conclude that a significant increase in percent cell migration rate and MMP-9 levels in the presence of bone under dynamic conditions is not solely regulated by CXCR4 but also results from the synergistic effect of *α*_v_ *β*_3_ integrins activation (figure 6(D)). Overall, our results suggest that mechanical cues by fluid flow play a significant role in the migration of prostate cancer cells.

## 5. Conclusion

Our findings suggest that interstitial flow-induced shear stress could be a critical factor in regulating the migration of prostate cancer cells at their extravasation stage. In the present study, we observed that the fluid flow rate corresponding to the physiological velocity of the interstitial fluid is optimum for better cell growth compared to the high flow rate, where we observed suppression in the growth rate of PC3 cells due to induction in apoptosis. Thus, we investigated the effect of physiological fluid velocity on the migration rate of PC3 cells and demonstrated that fluid shear stress contributed to an increased migration rate of PC3 cells via increased expression of *α*_v_ *β*_3_ integrins that further activate downstream signaling leading to an increase in MMP-9 levels. It is well accepted that fluid flow-derived shear stress is a fundamental determinant of cell behavior regulating tumor biology. Thus, our results are important for new therapies for controlling tumor progression and developing new inhibitors. The novel bioreactor described here could be utilized for understanding the fundamental mechanisms of growth and migration of different cancer types in the future.

## Data Availability

All data that support the findings of this study are included within the article (and any supplementary files).
